# Incidental Finding of Acute Appendicitis During Laparoscopic Cholecystectomy for an Acute Calculous Cholecystitis

**DOI:** 10.7759/cureus.21973

**Published:** 2022-02-07

**Authors:** Mansour Alkhurmudi, Bandar Ali, Abdullah Alzaharani

**Affiliations:** 1 General Surgery, Prince Sultan Military Medical City, Riyadh, SAU

**Keywords:** laparoscopy, incidental finding, simultaneous, appendicitis, cholecystitis

## Abstract

Acute cholecystitis and appendicitis are among the most common conditions encountered by general surgeons; however, they are rarely described simultaneously. We are reporting a rare incidental finding of early acute appendicitis during laparoscopic cholecystectomy for a 36-year-old lady who presented to our emergency department with signs and symptoms of acute calculous cholecystitis. Laparoscopy has proved to be a highly useful and ideal tool in the diagnosis and treatment of such surgical situations.

## Introduction

Acute appendicitis and acute cholecystitis are among the most common causes of acute abdominal pain, which is commonly due to a single pathology; however, clinicians need to be aware that multiple pathologies can rarely coexist.

Although rarely described, secondary appendicitis can occur together with other intra-abdominal inflammatory pathologies when the appendix lies close to the site of inflammation or inflammatory collection [1].

Our aim of this study is to highlight the importance of utilizing laparoscopic exploration of the entire abdomen in all cases presenting with an acute surgical abdomen, even if the initial diagnosis shows a single pathology, as multiple pathologies can be coincidentally discovered.

## Case presentation

A 36-year-old woman, medically and surgically free, presented to the emergency department with a two-day history of a new-onset right upper quadrant pain radiating to the back. The abdominal pain was described as severe and sharp. There was no history of nausea, vomiting, anorexia, fever, jaundice, change in bowel habits, or urinary symptoms.

Upon evaluation, the patient was in mild pain and hemodynamically stable (no fever or tachycardia). Her abdominal examination was significant for epigastric tenderness as well as a positive Murphy’s sign. She has normal bowel sounds and no palpable masses or organomegaly.

**Figure 1:**
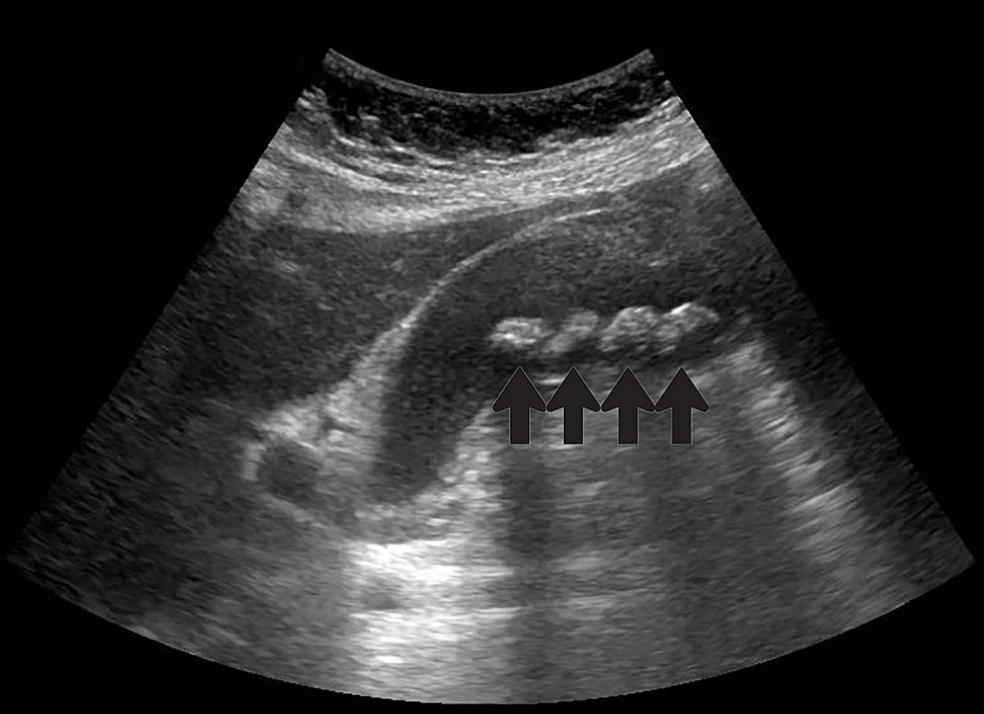
US gallbladder left lateral decubitus view with black arrows indicating multiple stones

**Figure 2:**
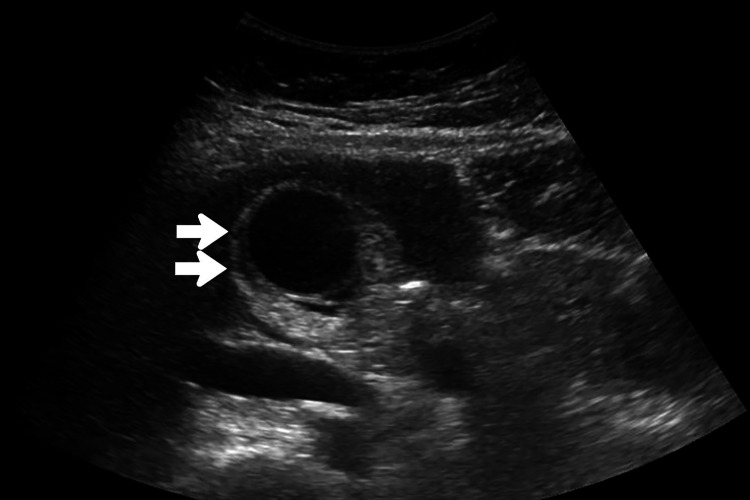
US gallbladder transverse view with white arrows showing thickened wall

A blood workup showed no leukocytosis (WBC 7.54 × 109/L), a normal liver function test (alkaline phosphatase 68 units/L, alanine aminotransferase 15 units/L, total bilirubin 10.4 mcmol/L), and a normal renal function test and amylase level. US abdomen showed multiple gallbladder stones associated with a trace amount of pericholecystic fluid, gallbladder wall thickening (6.3 mm), and a normal common bile duct (CBD) caliber of 0.4 cm (Figures 1, 2).

An impression of acute calculous cholecystitis was made. The patient was hydrated with IV fluids, a second-generation cephalosporin (Cefuroxime 750 mg IV TID) was started, and the patient was taken the next day for laparoscopic cholecystectomy under general anesthesia.

**Figure 3:**
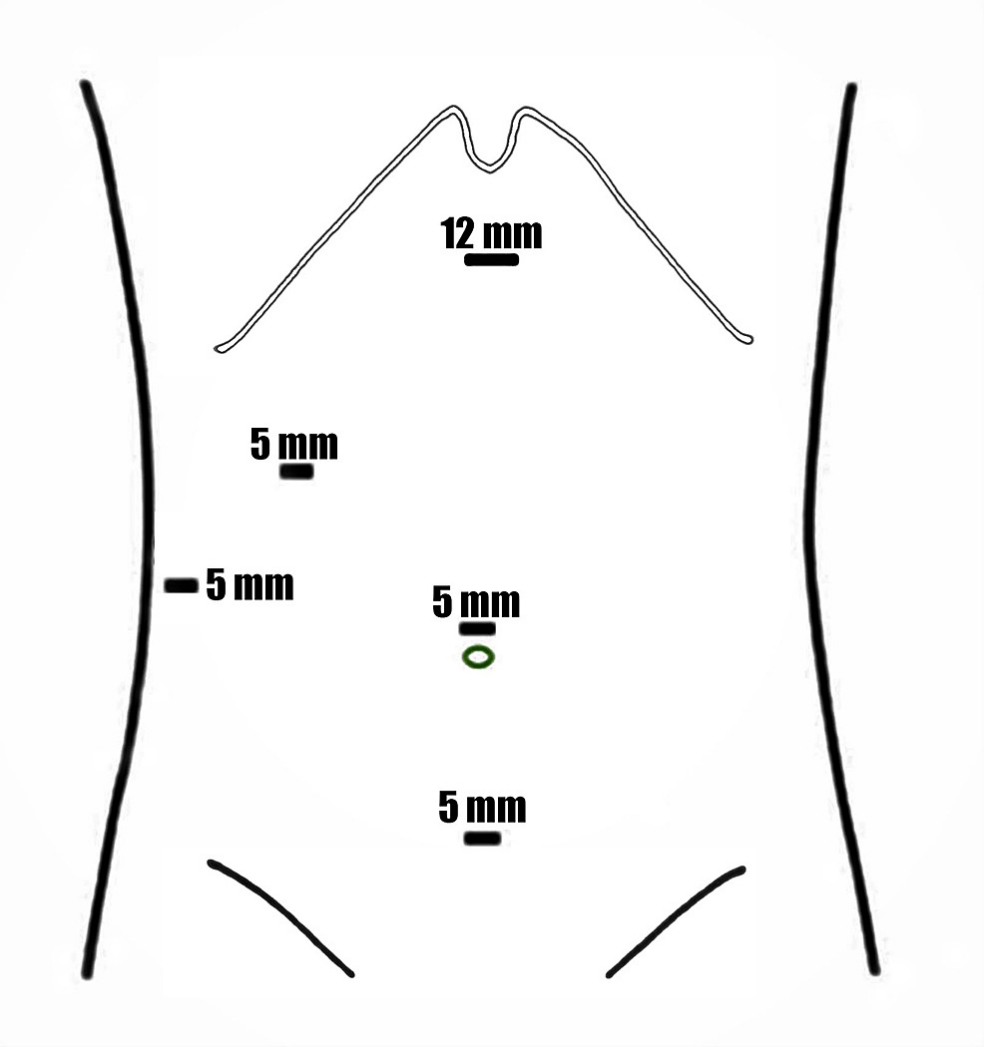
Ports placement

**Figure 4:**
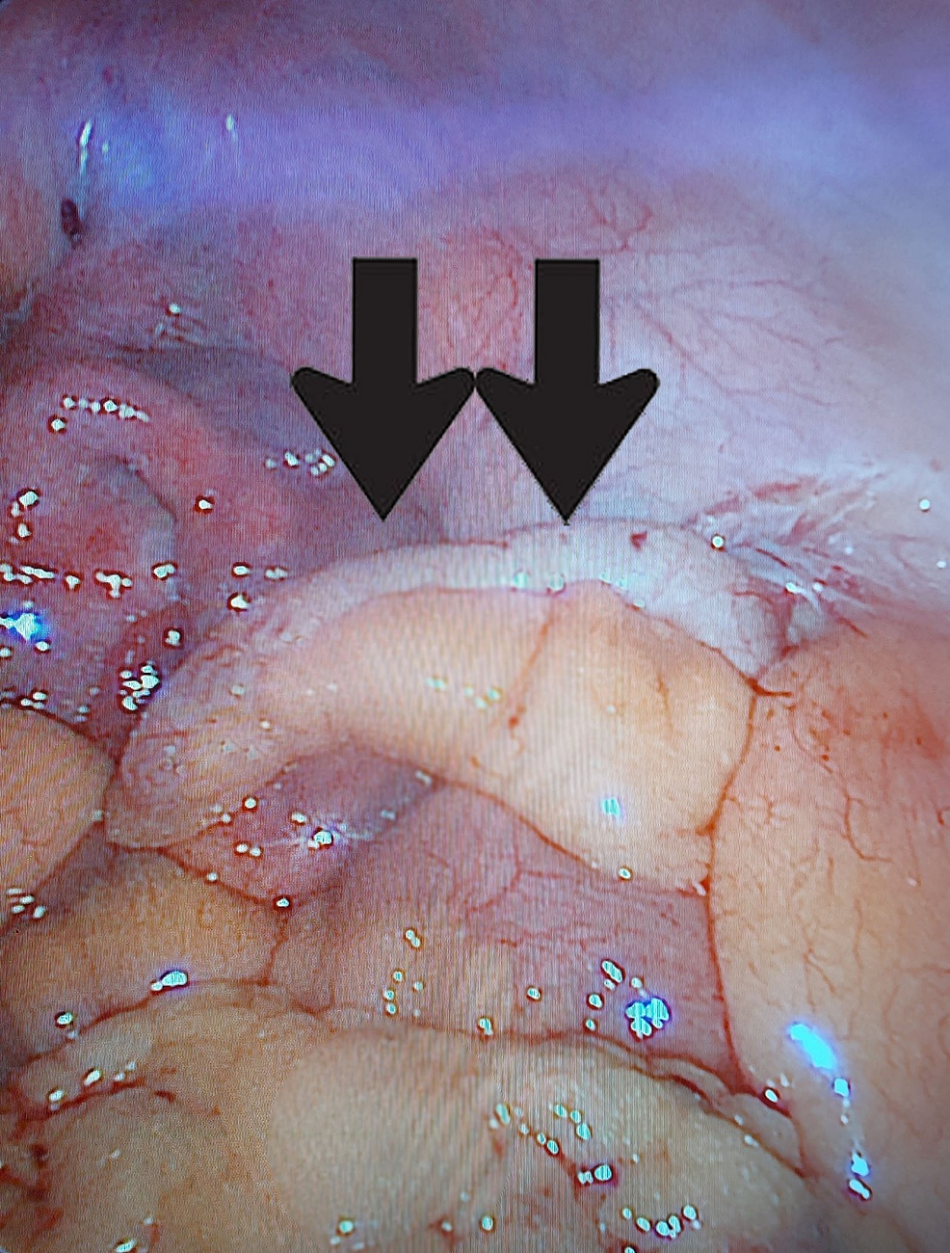
Intra-operative finding of inflamed appendix (black arrows)

The abdomen was insufflated using a veress needle at the Palmers’ point, then a 5 mm supra-umbilical port was introduced under camera guidance, and an additional three ports were inserted under vision (one 12 mm port and two 5 mm ports) (Figure 3). Diagnostic laparoscopy revealed a minimal amount of pericholecystic fluid collection with free fluid (light brownish yellow) extending along the right paracolic gutter down to the pelvis. The gallbladder was found to be edematous, severely inflamed, and distended with omental adhesions. The appendix was located in the pelvis surrounded by the inflammatory fluids and it was turgid and edematous with a thickened mesoappendix and serosal hyperemia (Figure 4). The rest of the GIT was normal. Aspiration of the gallbladder was carried out to enable adequate grasper function; adhesions were released, and cholecystectomy was performed. Another suprapubic 5 mm port was inserted (Figure 3), and an appendectomy was performed. A drain was inserted, extending from the subhepatic area down to the pelvis along the right paracolic gutter.

The postoperative course was uneventful; drain output was minimal in amount and haemoserous. All the postoperative blood workup was normal. The patient regained full oral intake, passed bowel motion, and was discharged on post-op day 2 after drain removal. Histopathology confirmed the diagnosis of acute calculous cholecystitis and acute appendicitis (dilated lumen, filled with fecalith material and early acute inflammation).

## Discussion

Simultaneously discovered appendicitis and acute cholecystitis is a rare situation, with only a few cases reported in the literature. A recent review by Buhamed et al. [2] found 11 case reports of co-existent acute appendicitis and acute cholecystitis, and four cases presented with right upper quadrant abdominal pain, which was similar to our patient’s main complaint.

As noted in the previous literature review, variable acute presentation of both appendicitis and cholecystitis at the same time can be difficult to diagnose as patients may present with either right upper quadrant pain, right-sided or diffuse abdominal pain. However, the typical presentation of acute appendicitis is an initial peri-umbilical pain that shifts to the right lower quadrant area [3]. Migratory pain is an important feature in the patient's history, with a reported sensitivity and specificity of 80% [4]. Patients with acute appendicitis usually have one or more of the following signs: rebound tenderness (pain on palpation over McBurney’s point), Rovsing, Psoas, and Dunphy signs. However, their absence should not be used to exclude acute appendicitis [3].

Patients with acute cholecystitis commonly present with a history of pain in the right upper quadrant and/or epigastric areas along with a positive Murphy's sign (high sensitivity for acute cholecystitis [5-7]) on physical examination.

In our case, interestingly, the patient presented with signs and symptoms that were only related to the acute calculous cholecystitis (which can be explained by the final histopathology of her appendix, which showed early acute inflammatory changes).

Few theories to explain the possible cause of concurrent inflammation of more than one intra-abdominal organ, one of these (although rarely described) is that secondary appendicitis can develop in conjunction with other intra-abdominal inflammatory pathologies when the appendix lies adjacent to the site of inflammation or inflammatory collection [1].

On the other hand, one hypothesis that can explain the occurrence of acute cholecystitis secondary to appendicitis is the result of direct invasion or translocation of bacteria from the muscularis propria of a gangrenous appendix into the portal venous system, which can lead to impaired bile salt excretion and contamination of the gallbladder bile, causing acute cholecystitis [2]. This hypothesis is supported by the incidence of hyperbilirubinemia in acute appendicitis [8].

Incidental findings discovered during surgery result in ethical and legal dilemmas for the surgeon [9], mainly because no specific consent was obtained from the patient, and in such a situation, treatment of any unexpected pathology should be in the patient’s medical interests.

## Conclusions

Acute abdomen in most cases is related to a single pathology. However, surgeons should be aware that multiple incidental pathologies can be found during intra-operative exploration. Secondary appendicitis discovered in our case can be explained by the presence of the appendix adjacent to the inflamed collection caused by the severely inflamed gallbladder, or it could be a coincidental finding, and they were unrelated to each other.

The laparoscopic approach allows surgical access to explore the entire abdomen, avoiding multiple open incisions or missing other causes of the surgical abdomen that can lead to the patient’s persistent pain, diagnosis confusion, and the need for further surgical exploration.
